# *FRG3*, a Target of slmiR482e-3p, Provides Resistance against the Fungal Pathogen *Fusarium oxysporum* in Tomato

**DOI:** 10.3389/fpls.2018.00026

**Published:** 2018-01-25

**Authors:** Hui-Min Ji, Min Zhao, Ying Gao, Xin-Xin Cao, Hui-Ying Mao, Yi Zhou, Wen-Yu Fan, Katherine A. Borkovich, Shou-Qiang Ouyang, Peng Liu

**Affiliations:** ^1^College of Horticulture and Plant Protection, Yangzhou University, Yangzhou, China; ^2^Department of Plant Pathology and Microbiology, Institute for Integrative Genome Biology, University of California, Riverside, Riverside, CA, United States; ^3^Joint International Research Laboratory of Agriculture and Agri-Product Safety of Ministry of Education of China, Yangzhou University, Yangzhou, China; ^4^Key Laboratory of Plant Functional Genomics of the Ministry of Education, Yangzhou University, Yangzhou, China; ^5^Testing Center, Yangzhou University, Yangzhou, China

**Keywords:** NBS-LRR, microRNA, *FRG3*, *Fusarium oxysporum*, wilt disease, disease resistance, tomato

## Abstract

The vast majority of plant disease resistance (*R*) genes encode nucleotide binding site–leucine-rich repeat (NBS-LRR) proteins, which specifically determine the plant immune response and have been demonstrated to be targets of several microRNA (miRNA) families. The fungus *Fusarium oxysporum* f. sp. *lycopersici* (FOL) causes vascular wilt disease in tomato worldwide. Here, we explored a possible role for *FGR3* in tomato defense against FOL. *FRG3* is a predicted NBS-LRR like gene that is targeted by slmiR482e-3p, a member of slmiR482 miRNA family. Northern blot data demonstrated that all seven members of the slmiR482 family were regulated in diverse ways after infection by FOL. The ability of *FRG3* to be regulated by slmiR482e-3p was confirmed at the transcript level by co-expression studies in *Nicotiana benthamiana*. A virus-induced gene silencing (VIGS) approach revealed that *FRG3* confers resistance to the Motelle tomato cultivar. Taken together, our study has identified a novel *R* gene, *FRG3*, which is targeted by slmiR482e-3p at the transcript level, and is necessary for resistance to tomato wilt disease *in planta*.

## Introduction

Plant defense against pathogenic microorganisms is based on two major layered innate immune systems. Upon exposure to pathogen invasion, recognition of microbe-associated molecular patterns (MAMPs) by pattern-recognition receptors (PRRs) in the plant leads to a general defense response referred to pathogen-associated molecular patterns (PAMP)-triggered immunity (PTI), also known as basal defense. The PTI response includes altered production of reactive oxygen species (ROS), hormone and metabolite levels, deposition of calluses, and accumulation of pathogenesis related proteins (PRs) (Jones and Dangl, 2006). In return, pathogens have evolved effectors to sabotage PTI. Plants have acquired disease resistance (*R*) genes to recognize the presence or action of specific effectors, directly or indirectly, and active effector-triggered immunity (ETI), which is a fast and strong form of immunity. This innate immune system is referred to the second defense (Jones and Dangl, 2006; Fei et al., 2016).

There is accumulating evidence that microRNAs (miRNAs) are involved in regulating plant immunity (Fei et al., 2016). MiRNAs, single-stranded RNA molecules of ∼20–24 nucleotides in length, are endogenously processed from single-stranded non-coding RNA species (Reinhart et al., 2000; Llave et al., 2002). It is well known that plant miRNAs play vital roles in multiple biological processes, including plant development, hormone signaling and biotic/abiotic stress responses, primarily acting on their target mRNAs through cleavage or translation repression (Aukerman and Sakai, 2003; Palatnik et al., 2003; Bartel, 2004; Sunkar and Zhu, 2004; Sunkar et al., 2006, 2007; Felippes et al., 2008; Padmanabhan et al., 2009; Rubio-Somoza et al., 2009). Recent work has demonstrated that host endogenous miRNAs function to counter-act pathogens as well. For example, many miRNA families, such as the miRNA482/2118 superfamily, target nucleotide-binding site and leucine-rich repeat domain containing proteins (NBS-LRRs) (Zhai et al., 2011; Li et al., 2012; Shivaprasad et al., 2012; Ouyang et al., 2014; Xia et al., 2015). The NSB-LRR gene family is one of at least five different classes of *R* genes identified to date, and also represents the *R* gene class with the most members (Van Ooijen et al., 2007). Most NBS-LRR genes are organized in clusters in the plant genome and the number of NBS-LRRs in a plant species is indicative of the reservoir of proteins available for the response to effectors (Kuang et al., 2004).

Our previous study reported that slmiR482f (referred to as slmiR482e-3p) and slmiR5300, two members of the miR482/2118 superfamily, regulate resistance to *Fusarium oxysporum* f. sp. *lycopersici* (race 2) (FOL) in tomato by targeting *NBS-LRR* genes (Ouyang et al., 2014). Furthermore, miR482a targets mRNAs for *R* genes with NBS-LRR motifs by degrading mRNAs directly and through generation of secondary small interfering RNAs (siRNAs) in *Nicotiana benthamiana* infected with *Pst* DC3000 (Li et al., 2012; Shivaprasad et al., 2012).

*Fusarium oxysporum* is a biotrophic pathogen that is the causal agent of plant wilt disease. Accumulating data indicate that *F. oxysporum* is a large species complex, with more than 150 host-specific forms causing disease in vegetables, fruit trees, wheat, corn, cotton and ornamental crops (Di Pietro and Roncero, 1998; Leslie and Summerell, 2006). FOL infects vascular bundles in the plant host, leading to clogged vessels, yellowing of leaves, wilting and finally death of the whole plant. Three physiological races of FOL have been distinguished based on their specific pathogenicity toward tomato cultivars (Di Pietro and Roncero, 1998; Kawabe et al., 2005; Leslie and Summerell, 2006; Takken and Rep, 2010).

Tomato (*Solanum lycopersicum*) is a worldwide economic crop and has also been studied as a model plant for the molecular basis of resistance mechanisms. Four *R* genes for resistance to FOL have been discovered from wild tomato species, including the *I* and *I2* genes from *S. pimpinellifolium*, and the *I3* and *I7* gene from *S. pennellii*. Among these four R genes, *I2, I3*, and *I7* have been cloned, and shown to encode NBS-LRR proteins (Ori et al., 1997; Simons et al., 1998; Catanzariti et al., 2015; Gonzalez-Cendales et al., 2016). Previous work has demonstrated that the *I2* and *I3* genes confer resistance to race 2 and race 3 lines of FOL, respectively (Simons et al., 1998; Catanzariti et al., 2015). The *I2* locus encodes an R protein that recognizes the *avr2* gene product from FOL (race 2) (Houterman et al., 2009). *I3* encodes an S-receptor-like kinase (SRLK) gene that confers Avr3-dependent resistance to FOL (race 3) (Catanzariti et al., 2015).

Previously, we utilized two near-isogenic tomato cultivars, susceptible Moneymaker (*i2*/*i2*) and resistant Motelle (*I2*/*I2*), to study the interaction between tomato and FOL (Ouyang et al., 2014). The genotypes of these two tomato cultivars differ at *I2* and in their response to FOL infection (Ori et al., 1997; de Ilarduya et al., 2001; Yu and Zou, 2008). During that study, we identified and characterized four *R* genes encoding NB domain-containing proteins that were required for full resistance to FOL in tomato (Ouyang et al., 2014).

In this study, we demonstrate that the slmiR482 family responds to FOL invasion in different tomato cultivars. We further showed that *FRG3*, encoding a prospective NBS-type R protein, is targeted by slmiR482e-3p, and contributes to tomato innate immunity against FOL.

## Materials and Methods

### Tomato Lines and Inoculation with FOL Cultures

Two tomato near-isogenic cultivars (cv.), the resistant Motelle (Mot, *I2*/*I2*) and susceptible Moneymaker (MM, *i2*/*i2*), described previously, were used in this study (Ouyang et al., 2014). Tomato seedlings growing at 25°C with a 16/8-h light/dark cycle for 2 weeks were used for all experiments. The wild-type *F. oxysporum* f. sp *lycopersici* (race 2) (FOL) strain was FGSC 9935. Two-week-old tomato seedlings were removed from soil and roots incubated in a solution of FOL conidia at a concentration of 1 × 10^8^/ml for 30 min. Water-treated tomato seedlings were used as the negative control. Forty seedlings were used for each treatment. Plants were then replanted in soil and maintained in a green house at 25°C for 24 h with constant light as described previously (Ouyang et al., 2014). Plants were removed from soil, and roots were rinsed gently and excised, then immediately frozen in liquid nitrogen and stored at -80°C. In order to control for experimental variation, all experiments were repeated three times.

### Northern Blot Analysis and Quantitative RT-PCR

Total RNA was isolated from roots using TRIzol^®^ Reagent (#15596026, Life Technologies, CA, United States) according to the manufacturer’s recommendations. For miRNA Northern blot analysis, 20 μg of total tomato root RNA was resolved using urea polyacrylamide gel electrophoresis (PAGE). MiRNA-specific oligonucleotide probes (**Table 1**) were end-labeled using γ-32P-ATP (#M0201, New England Biolabs, Ipswich, MA, United States). Blots were stripped and reprobed using at most one additional miRNA probe. The upper section of the blot was used for the loading control using a U6 oligonucleotide probe. All blots were imaged using a PhosphorImager (Molecular Dynamics/GE Life Sciences, Pittsburgh, PA, United States) and band intensities quantified using Imagequant software.

**Table 1:** Primers used in this study.

Primer Name	Application	Sequence (5′ – 3′)
slmiR482a	Northern Blot Probe	TAGGAATGGGTGGAATTGGAAA
slmiR482b	″	GGCATGGGCGGTGTAGGCAAGA
slmiR482c	″	GGCATGGGCGGTGTAGGCAAGA
slmiR482d-5p	″	TTTTTCCATCCCACCCACTCC
slmiR482d-3p	″	TTGGCATGGGTGGAATAGGAAA
slmiR482e-5p	″	AATCTTTCCACCCCACCCACA
slmiR482e-3p	″	GGTATGGGAGGAGTAGGAAAGA
U6	″	GGGGCCATGCTAATCTTCTCTG
Solyc12g099060-F		CAAAACAACAGTTGCCCAGC
Solyc12g099060-R	″	GAAAGAATGCCTTGAGTACGC
Sl18S-rRNA-F	″	TGACGGAGAATTAGGGTTCG
Sl18S-rRNA-R	″	CCTCCAATGGATCCTCGTTA
Solyc12g099060-F	q-PCR Primer	CAAAACAACAGTTGCCCAGC
Solyc12g099060-R	″	GAAAGAATGCCTTGAGTACGC
*Mi-1*-F	″	GAAACAACTGTCATTGCAT
*Mi-1*-R	″	GAAACAACTGTCATTGCAT
Mot-*I2*-F	″	CCTCCTTTTCTCACCTCACT
Mot-*I2*-R	″	CAATCGATATTTATGATGGG
Nt-Actin-F	″	GAAAACTGGACAGAACTG
Nt-Actin-R	″	CATCCTTGAGGCTCATTCG
IGS1049	″	TGCGATTTGGACGAGATATGTG
IGS1050	″	ATTTGCCTACCCTGTACCTACC
Solyc12g099060-attB1	VIGS Vector Construction	GGGGACAAGTTTGTACAAAAAAGCAGGCTGCAATCTCGTGAATCAGAGAATGTG
Solyc12g099060-attB2	″	GGGGACCACTTTGTACAAGAAAGCTGGGTCACATTCTCTGATTCACGAGATTGC
slmiR166a-F	Co-expression Vector Construction	TTAAGAATTCGTTGAAGTCAAGCTAAGATGCG
slmiR166a-R	″	TTAAACTAGTCGAAACAAGTTTTAGTAGGTGCC
slmiR482e-3p-F	″	TTAAGAATTCCGGTTAGATCCAAGTTCTGG
slmiR482e-3p-R	″	TTAAACTAGTATAATGTAACCCCACCGACC
Solyc12g099060-F	″	TTAAGAATTCATGGGCGTGAAGGAGGAGCT
Solyc12g099060-R	″	TTAAACTAGTACGTCCTGCCACATTCAGCT

Transcript levels for *FRG3* were determined using both Northern blot analysis and quantitative reverse transcriptase PCR (qRT-PCR). For Northern analysis, 10 μg of total RNA was resolved on 1% agarose gels and processed as described previously (Ouyang et al., 2014). Probe templates were prepared by amplification of cDNA using specific primers in PCRs (**Table 1**). Probes were labeled using the random priming method according to the manufacturer’s protocol (#U1100, Promega, San Luis Obispo, CA, United States). All blots were stripped and reprobed using *18S RNA* probe as a loading control. Blots were imaged and band intensities quantitated as described above.

For qRT-PCR analysis, 1 μg of total RNA was used for first strand cDNA synthesis with Murine Leukemia Virus Reverse Transcriptase (M-MLV; #4368813, Life Technologies, Grand Island, NY, United States). Diluted cDNA was employed as the template for qRT-PCR (iQ5, Bio-Rad, Philadelphia, PA, United States) using Action as internal control. *FRG3* gene-specific primers are listed in **Table 1**.

### Transient Expression of MicroRNAs and *FRG3* in *Nicotiana benthamiana* and Target Validation

DNA fragments encoding sly-miR482d-3p and sly-miR482e-3p and the target gene *FRG3* were inserted into vector GATEPEG100. All constructs were transformed into *Agrobacterium tumefaciens* strain GV3101. *N. benthamiana* plants were maintained in a green house at 25°C with a 16/8-h light/dark cycle. *A. tumefaciens* cultures were grown in liquid LB medium with selection (Qiao et al., 2013). All constructs were co-infiltrated into *N. benthamiana* leaves. After 40 h, infiltrated leaves were harvested, and cellular protein was extracted (Kocken et al., 1993). Western blot analysis with Anti-FLAG Antibody (#635691, Clontech, Mountain View, CA, United States) was conducted as described previously (Ouyang et al., 2014).

MiRNA-target validation was performed using a 5′ RACE assay (Ouyang et al., 2014). Briefly, total RNA was isolated from infiltrated *N. benthamiana* leaves. The Poly (A^+^) mRNA fraction was directly ligated using an RNA Oligo adaptor. Reverse transcription was performed, followed by semi-quantitative PCR using gene-specific primers (see **Table 1**). PCR products were cloned into the pGEM-T Easy Vector (#A1360, Promega) and sequenced.

### Virus-Induced Gene Silencing (VIGS) Constructs and Phenotype Assessment

Virus-induced gene silencing was utilized to suppress expression of *FRG3* using TRV-based vectors (pTRV1 and pTRV2) (Ouyang et al., 2014). Briefly, the 3-UTR of *FRG3* was amplified using gene-specific primers (**Table 1**) and cloned into the pTRV2 vector. Vectors for silencing of the Phytoene Desaturase (*PDS*) gene were used as a positive control (Mantelin et al., 2011). Four weeks after infiltration, leaves were harvested, RNA isolated, and the degree of silencing determined using qRT-PCR. The same plants were then infected with FOL or water for phenotypic analysis. Disease symptoms of VIGS plants were assessed after four more weeks. Genomic DNA was isolated from leaves and used for determining relative levels of FOL using qPCR of the rRNA intergenic spacer region (IGS) (Validov et al., 2011).

### Phylogenetic Analysis

A phylogenetic analysis was carried out for the six members from the protein I2 family combining with four NBS-LRR proteins which were reported by our group previously (Ouyang et al., 2014). All amino acid sequences were obtained from the Sol Genomics database^1^. All sequences were aligned using ClustalW. The evolutionary history was inferred by using the Maximum Likelihood method based on the JTT matrix-based model (Jones et al., 1992). The percentage of trees in which the associated taxa clustered together is shown next to the branches. Initial tree for the heuristic search were obtained automatically by applying Neighbor-Join and BioNJ algorithms to a matrix of pairwise distances estimated using a JTT model, and then selecting the topology with superior log likelihood value. Point accepted mutation (PAM) was used for an amino acid transition matrix. The tree is drawn to scale, with branch lengths measured in the number of substitutions per site. The analysis involved 12 amino acid sequences. All positions containing gaps and missing data were eliminated. There were a total of 204 positions in the final dataset. OsGAPDH was used as a root by midpoint method. Evolutionary analyses were conducted in MEGA7 (Kumar et al., 2016).

### Statistical Analysis

All data were subjected to Student’s *t*-test analysis by SPSS 11.5 (SPSS Company, Chicago, IL, United States).

## Results

### Characterization of the SlmiR482 Family during the Response to FOL in Tomato Roots

**FIGURE 1:**
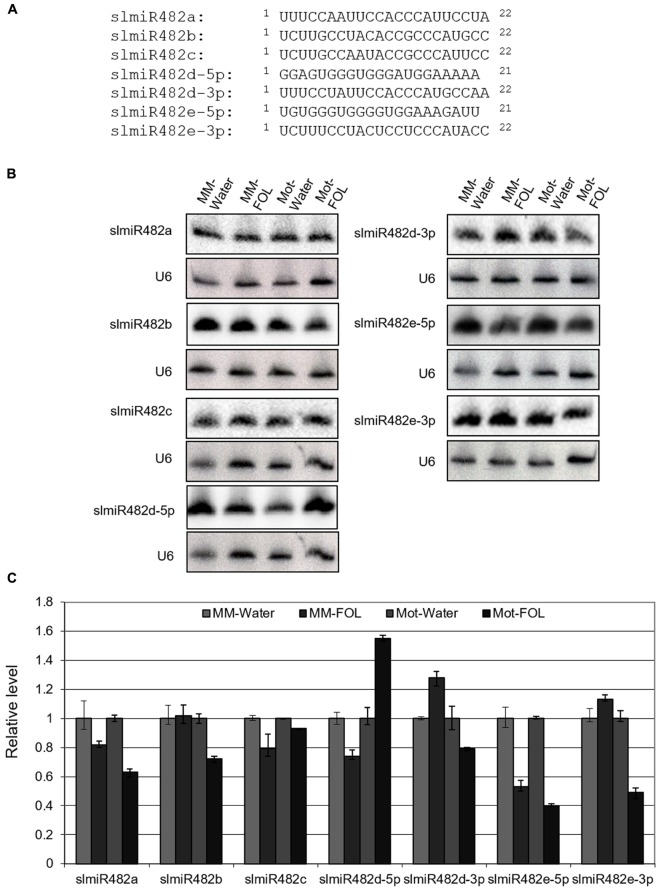
Responses of slmiR482 family members to FOL infection. **(A)** Alignment of slmiR482 family members. Sequences were aligned to maximize identical residues. **(B)** Northern blot analysis of slmiR482 family members. Root total RNA samples (20 μg) from Moneymaker treated with water (MM-water), Moneymaker infected with FOL (MM-FOL), Motelle treated with water (Mot-water) and Motelle infected with FOL (Mot-FOL) were used to prepare Northern blots. Oligonucleotide probes were used to quantitate levels of individual slmiR482 family members. Blots were probed with U6 to serve as a loading control. **(C)** Quantification of relative miRNA amounts. miRNA levels obtained from Northern analysis in **(B)** were normalized using water treatment as 100% (1.0).

MiRBase21 has seven miR482 entries including miR482a, b, c, d-5p, d-3p, e-5p, and e-3p of which five correspond to miR482s targeting NBS-LRRs and two correspond to the complementary miR^∗^ sequences (miRBase21, Kozomara and Griffiths-Jones, 2014). SlmiR482 is unusual among microRNA families, in that most members are 22 rather than 21 nucleotides, and have more sequence variability than other miRNA families (**Figure 1A**) (de Vries et al., 2015). To characterize the response of the slmiR482 family to FOL in tomato, we performed small RNA Northern blot analysis with specific probes for all seven members of the slmiR482 family. Interestingly, the RNA blot results revealed that all seven members were expressed and regulated differentially in two tomato cultivars plants after FOL infection, which differed from our previous sRNA-seq results (unpublished data) (**Figure 1B** and **Supplementary Figures S1–S7**). Levels of slmiR482e-5p were suppressed significantly in both Moneymaker and Motelle upon FOL infection. SlmiR482b, slmiR482d-3p, and slmiR482e-3p were down-regulated in Motelle, but, on the contrary, were up-regulated slightly in Moneymaker after FOL treatment. However, slmiR482d-5p presented the opposite pattern, with decreased levels in Moneymaker, and increased amounts in Motelle significantly, when treated with FOL (**Figures 1B,C**). These findings suggest that slmiR482 family members play different roles during the defense of tomato against the pathogen FOL.

Based on the results from both sRNA-seq and small RNA northern blot analysis, slmiR482d-3p and slmiR482e-3p were determined to be down-regulated in Motelle but upregulated in Moneymaker after infection, suggesting that both may negatively regulate levels of resistance gene mRNAs and/or their translation in tomato. Therefore, we focused our attention on the targets of slmiR482d-3p and slmiR482e-3p.

### A Predicted Target of SlmiR482e-3p Exhibits Altered Expression after Infection with FOL

**FIGURE 2:**
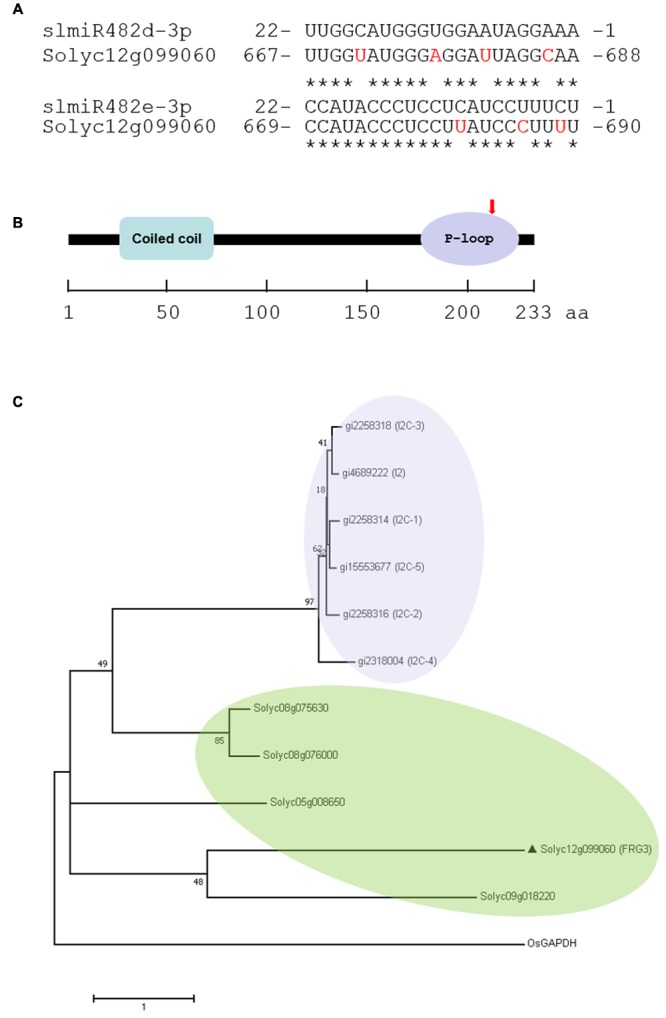
Prediction of slmiRNA targets and protein domain analysis. **(A)** Predicted mRNA target sequences and targeting site. Targets of slmiR482 family members were identified using the psRNATarget algorithm (http://plantgrn.noble.org/v1_psRNATarget/). Alignments were made using ClustalW (http://www.ebi.ac.uk/Tools/psa/). The nucleotides shown in red in the mRNA are mismatches with the corresponding miRNA. **(B)** Prediction of translated protein domains. Domain prediction was performed using Interpro (http://www.ebi.ac.uk/interpro/). CC: Coiled-Coil domain. The miRNA binding site is indicated with a red arrow in the P-loop region. **(C)** Phylogenetic analysis of Solyc12g099060 and I2 homologs. The Maximum Likelihood method based on the JTT matrix-based model. PAM was used for an amino acid transition matrix. OsGAPDH was used as a root by midpoint method. Evolutionary analyses were conducted in MEGA7. The sequences were including gi| 4689222 (I2), gi| 2258314 (I2C-1), gi| 2258316 (I2C-2), gi| 2258318 (I2C-3), gi| 2318004 (I2C-4), gi| 15553677 (I2C-5), Solyc08g075630, Solyc08g076000, Solyc09g018220, Solyc05g008650, Solyc12g099060 (FRG3) and OsGAPDH.

We utilized the psRNATarget algorithm (Dai and Zhao, 2011) to predict targets of the slmiR482 family. For each member, we found several potential targets in the tomato genome (**Supplementary Table S1**). Intriguingly, Solyc12g099060 was a putative target for both slmiR482d-3p and slmiR482e-3p, with a two-nucleotide shift in the binding site (**Figure 2A**). Solyc12g099060 is predicted to encode an NBS-type resistance protein containing coiled-coil (CC) and P-loop domains. The two miRNAs are predicted to bind in the P-loop region of the transcript (**Figure 2B**).

It is well known that the dominant *I2* locus in tomato, introgressed from the wild tomato species *S. pimpinellifolium*, confers resistance against FOL race 2 (Simons et al., 1998). Six homologs, including *I2, I2C-1, I2C-2, I2C-3, I2C-4*, and *I2C-5* were identified at the *I2* locus in tomato (Simons et al., 1998). To clarify the genetic homology between the *I2* family and Solyc12g099060, phylogenetic analysis was performed based on the amino acid sequences. Our results showed that the *I2* family clusters in a separate group from Solyc12g099060, as well as the four NBS-LRR genes that we studied previously, Solyc08g075630, Solyc08g076000, Solyc05g008650, and Solyc09g018220 (Ouyang et al., 2014). Solyc08g075630 and Solyc08g076000 were targeted by slmiR482e-3p (slmiR482f), and Solyc05g008650 and Solyc09g018220 were targets of slmiR5300. Surprisingly, Solyc12g099060 shares a more recent common ancestor with Solyc09g018220 than it does with the other targets of 482e-3p (**Figure 2C**).

### The Response of *FRG3* to FOL Infection in Tomato

**FIGURE 3:**
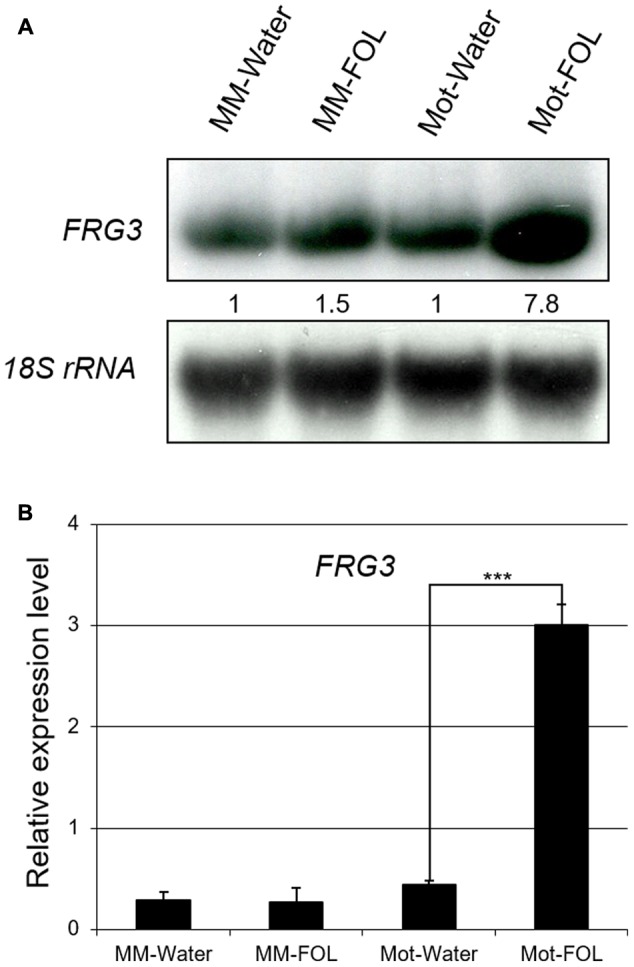
Expression of *FRG3* is induced in resistant Motelle after infection by FOL. **(A)** Northern blot analysis. Ten μg of total root RNA were used to prepare Northern blots. Blots were stripped and reprobed using an 18S RNA probe as a loading control. Blots were imaged and bands quantitated as described above. **(B)** qRT-PCR analysis. The total root RNA samples in **(A)** were used for qRT-PCR with *FRG3* primers using *actin* for normalization. ^∗∗∗^ indicate significant differences when compared to the corresponding control plants in the same treatments at *p* < 0.001.

To test the possibility that the presence of FOL would affect the expression of Solyc12g099060, we checked the transcript level of Solyc12g099060 under water/FOL treatments in both tomato varieties using both total RNA Northern blot analysis and quantitative RT-PCR. Basal expression levels of Solyc12g099060 mRNA were 1.5-fold higher in Motelle relative to Moneymaker (**Figure 3A**). Importantly, Solyc12g099060 mRNA was induced almost eightfold in Motelle after treatment with FOL, but only slightly increased in Moneymaker under the same conditions (**Figure 3A**). The Northern blot results (**Figure 3A** and **Supplementary Figure S8**) were consistent with those obtained using quantitative RT-PCR (**Figure 3B**). These findings support the conclusion that expression of Solyc12g099060 is induced by FOL infection in both tomato cultivars.

Based on the analysis above, we named Solyc12g099060 *FRG3* (FOL Resistance Gene 3).

### FRG3 Is Regulated by SlmiR482e-3p at the Transcriptional Level

To verify how the miRNA regulates the *FRG3* target, we implemented *Agrobacterium*-mediated transient co-expression experiments in *N. benthamiana*. *FRG3* and slmiRNAs were inserted into a binary construct containing a FLAG-tag (for *FRG3*). Vectors containing *FRG3* alone or slmiR166, which does not recognize *FRG3*, served as negative controls.

**FIGURE 4:**
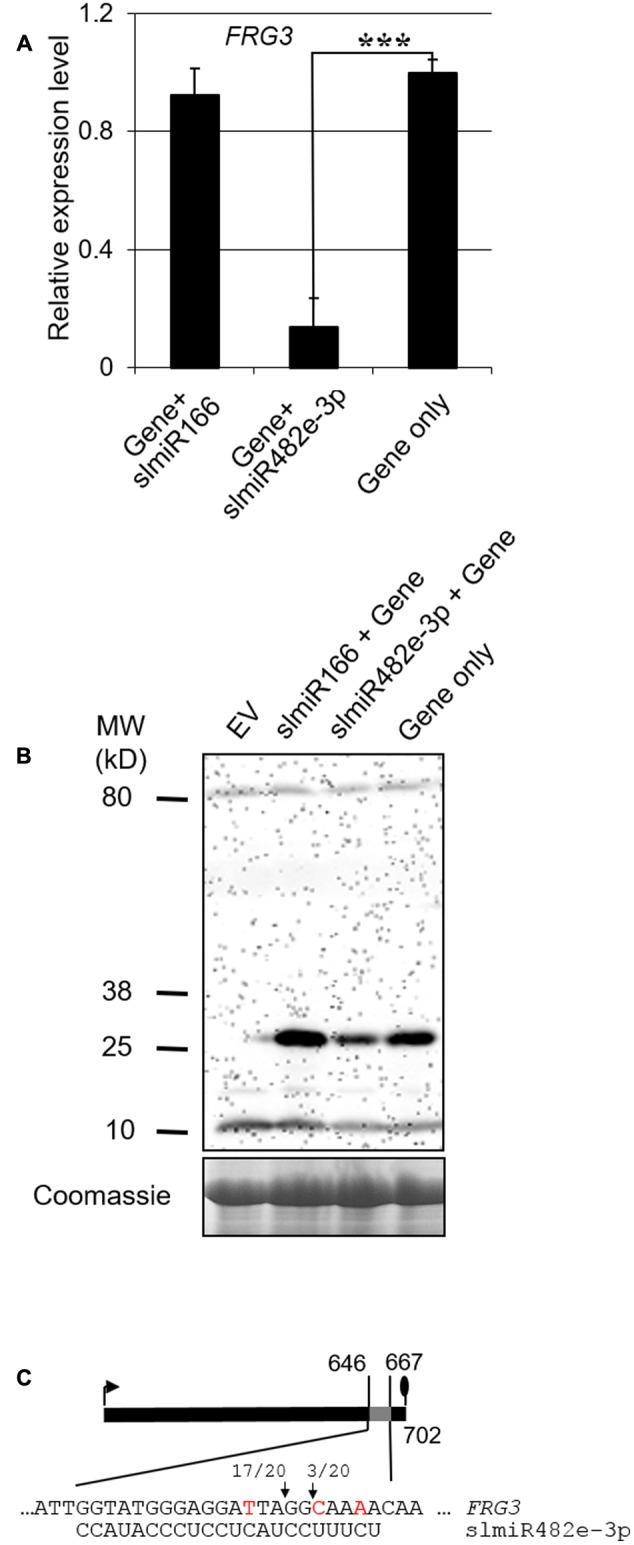
Validating the action of a slmiRNA on *FRG3* by transient co-expression in *Nicotiana benthamiana* leaves. **(A)** Quantification of *FRG3* transcript levels in total RNA isolated from *N. benthamiana* leaves using qRT-PCR. qRT-PCR was performed as described in the Section “Materials and Methods” using *FRG3* primers. Values were normalized to *N. benthamiana* actin. ^∗∗∗^ indicate significant differences when compared to the corresponding control plants in the same treatments at *p* < 0.001. **(B)** Determination of FRG3 protein levels. Crude total protein *(extracts isolated from *N. benthamiana* leaves infiltrated with different constructs were separated on SDS-PAGE gels and then used to prepare Western blots. Blots were reacted with a FLAG antiserum. A duplicate gel stained with Coomassie was used as loading control. Similar results were observed for three biological replicates. **(C)** Identification of the slmiRNA cleavage site on the target gene mRNA using 5′RACE. Total RNA samples were obtained as described above and subjected to 5′ RACE as described in the Section “Materials and Methods.” The arrows denote the detected cleavage sites, while the ratios indicate the fraction of events detected (out of 20 clones analyzed).)*

Total RNA was extracted from *N. benthamiana* leaves infiltrated with the *Agrobacterium* strains. We performed qRT-PCR to check the transcript level of *FRG3* during co-expression with a miRNA. In the presence of slmiR482e-3p, levels of the *FRG3* transcript were greatly decreased (**Figure 4A**). We then checked for possible translational control of FRG3 by the miRNA using Western blot analysis with antibody against the FLAG-tag. Our data showed that levels of FRG3 protein were down-regulated significantly by the presence of slmiR482e-3p (**Figure 4B**). Surprisingly, co-expression of *FRG3* and slmiR482d-3p in *N. benthamiana* did not lead to a detectable change in *FRG3* transcript or FRG3 protein (data not shown). These results strongly suggest that slmiR482e-3p, but not slmiR482d-3p, is responsible for the down-regulation of the *FRG3* target gene. The observation of suppressed *FRG3* mRNA levels is consistent with slmiR482e-3p acting on *FRG3* mainly at the transcript stability level.

To verify the cleavage site on the target mRNA, we performed RNA ligase mediated 5′ rapid amplification of cDNA ends (5′RACE) PCR analysis to detect the product of slmiR482e-3p mediated cleavage of *FRG3* mRNA after transient coexpression in *N. benthamiana*. Our results indicate that the major cleavage site in *FRG3* catalyzed by slmiR482e-3p occurred after the tenth nucleotide from the 5′ end of the miRNA (**Figure 4C**).

### Partial Silencing of *FRG3* Attenuates the Resistance of the Motelle Cultivar to FOL

To explore a possible role for *FRG3* in resistance to FOL, we employed a TRV-based VIGS system using the extreme 3′ end of the open reading frame (ORF) of *FRG3* and a portion of the 3′ untranslated region to knock-down the expression of *FRG3* in the resistant cultivar Motelle. As a positive control, Phytoene Desaturase (*PDS*) TRV-silenced plants (TRV-*PDS*) were generated in parallel (Mantelin et al., 2011). The photobleached phenotype was observed in TRV-*PDS* plants 4 weeks after TRV infection, signifying that silencing of the *PDS* gene had occurred. Therefore, all VIGS plants were treated with FOL 4 weeks after TRV infection. Motelle plants treated with water and Motelle plants transduced with empty TRV vector served as negative controls. Disease phenotypes were scored 4 weeks after FOL infection.

**FIGURE 5:**
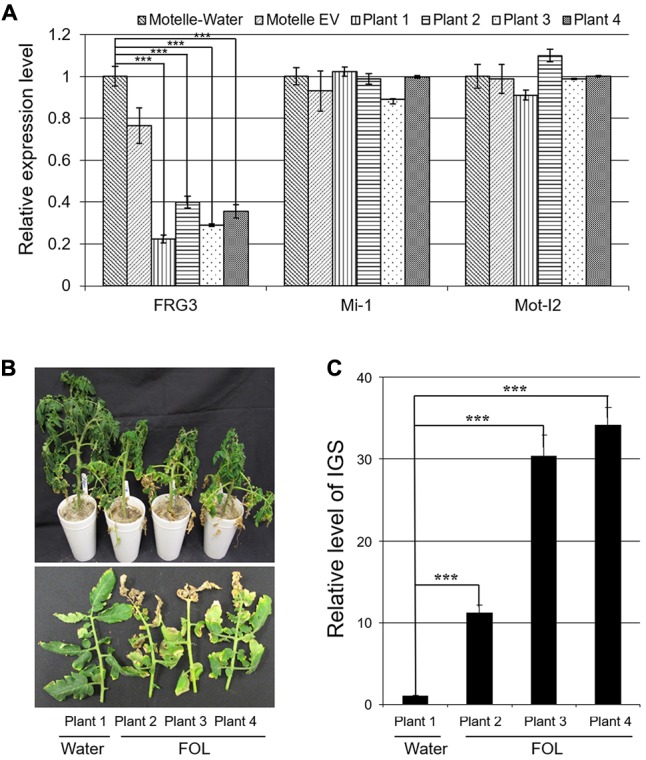
*FRG3* is required for full resistance of tomato cv. Motelle against FOL. Motelle plants were infiltrated with the *FRG3* knock-down construct (four plants) or empty vector (EV). **(A)** Assessing the degree of *FRG3* silencing using qRT-PCR. Leaflets were collected 4 weeks after VIGS. Total RNA was isolated and subjected to qRT-PCR to evaluate transcript levels of *FRG3*. *Mi-1* and *I2* levels were also analyzed in order to detect possible off-target effects of VIGS. Data were normalized to Actin. Errors are presented as the standard error. ^∗∗∗^ indicate significant differences when compared to the corresponding control plants in the same treatments at *p* < 0.001. **(B)** Wilt disease symptoms of VIGS tomato seedlings after infection with FOL. Tomato plants (Plants 2–4) were infected by FOL conidia at a concentration of 1 × 10^8^/ml 4 weeks after VIGS. Water treatment (Plant 1) was used as a negative control. Symptoms were analyzed 4 weeks later. **(C)** Determination of relative levels of FOL biomass in tomato leaves using qPCR. Genomic DNA was isolated from tomato leaves as described above. The Intergenic Spacer region (IGS) of FOL was used as a marker to assess relative fungal biomass. Errors are presented as the standard error. ^∗∗∗^ indicate significant differences when compared to the corresponding control plants in the same treatments at *p* < 0.001.

For all VIGS plants, transcript levels of *FRG3* were quantified by qRT-PCR prior to FOL infection. The data indicated that the mRNA level of *FRG3* was down-regulated by ∼60–80% in VIGS plants compared to control Motelle plants (**Figure 5A**). We also checked the mRNA level of *Mi-1* and *I2* to eliminate the possibility of off-target effects during VIGS (**Figure 5A**). All FOL-treated VIGS plants grew more slowly than control plants treated with water (Plant 1) and exhibited severe leaf wilting discoloration disease symptoms (**Figure 5B**; Plants 2–4).

To estimate the biomass of FOL in infected VIGS plants, we amplified the rRNA IGS from genomic DNA isolated from tomato leaves using qPCR. Our data demonstrates that FOL levels were elevated significantly in inoculated *FRG3* VIGS plants, particularly in Plants 2 and 3 (**Figure 5C**). This result is consistent with observed disease severity symptoms.

## Discussion

In the present study, we explored a possible function for the NBS-LRR type gene *FRG3* during FOL infection. *FRG3* is targeted by slmiR482e-3p, a member of slmiR482 family. The miR482 family is a subfamily of the miR482/2118 superfamily in plants, characterized by high sequence diversity among its family members (de Vries et al., 2015). Members of the miR482/2118 superfamily target the P-loop motif in NBS-LRR gene mRNAs (Shivaprasad et al., 2012). Our Northern blot results showed that all five slmiR482 family entries and two corresponding to the complementary miR^∗^ sequences found in miRBase 21 (Kozomara and Griffiths-Jones, 2014) displayed different responses to FOL infection in two tomato cultivars: susceptible Moneymaker and resistant Motelle. We only used those sequences provided by miRBase21with entries as miR482, distinguish between miR482 sequences and their complementary miR^∗^ sequences and provide the results from the target prediction for each of the seven miRNAs published as miR482/2118 members in miRBase21 (Dai and Zhao, 2011). Our previous study revealed that some biotic and abiotic stress-associated miRNAs, such as slmiR482 family and slmiR398, were suppressed in the resistant tomato cultivar Motelle after FOL treatment (Ouyang et al., 2014). In addition to conferring plant immunity by regulating the expression of target genes, miR482e can negatively regulate susceptibility to *Verticillium dahliae* infection in potato (*S. tuberosum*) (Yang et al., 2015). Moreover, transgenic expression of miR482 causes significant increases of nodule numbers in soybean (Li et al., 2010).

Our previous results demonstrated that slmiR482f (slmiR482e-3p) and slmiR5300, two members of miR482/2118 superfamily, acted on several NBS-LRR targets at either the transcript stability or translational level in tomato (Ouyang et al., 2014). In this study, data from our transient co-expression experiments in *N. benthamiana* indicated that slmiR482e-3p regulates *FRG3* at the transcript stability level. Targeting of the NBS-LRR mRNA can lead to the production of phased secondary small interfering RNAs (phasiRNAs), which activate a regulatory cascade by targeting the original and other NBS-LRR genes, resulting in translation suppression in many instances (Zhai et al., 2011; Shivaprasad et al., 2012). In our case, however, no phasiRNAs were predicted in the tomato genome (data not shown), perhaps due to *FRG3* playing a different role during the response to FOL invasion.

Motelle (resistant, *I2*/*I2*) and Moneymaker (susceptible, *i2*/*i2*) show different immune response to FOL (Di Pietro and Roncero, 1998; de Ilarduya et al., 2001; Yu and Zou, 2008). The *I2* gene in tomato encodes a coiled-coil (CC) NB-LRR protein that recognizes Avr2 produced from FOL (race 2) (Simons et al., 1998; Houterman et al., 2009). *I2* homologs have also been found in potato (Huang et al., 2005; Li et al., 2011) and pepper (Grube et al., 2000). The miR482 family was found to target *I2* homologs in potato (Li et al., 2012). However, no cleavage of *I2* homologs by miR482 has been observed in tomato. Phylogenetic analysis showed that *FRG3* was not a homolog of the *I2* family, leading us to propose that *FRG3* acts as a disease resistance partner to compensate the potential cost of *I2* homolog expression to fitness in tomato.

Plant immune responses can be activated rapidly by pathogen invasion. Our data demonstrates that *FRG3* is induced significantly and quickly (24 h after inoculation) in resistant Motelle after FOL infection. As expected, partial silencing of *FRG3* resulted in susceptibility of Motelle to FOL, along with enhancement of FOL biomass accumulation. The phenotypes of *FRG3* VIGS Motelle plants were not as severe as those observed in the susceptible Moneymaker after FOL infection, suggesting that knock-down of *FRG3* is not sufficient to abolish the effective disease resistance in Motelle.

Since the first miRNA (miR472) targeting NBS-LRR resistance genes was identified in *Arabidopsis* (Lu et al., 2005), more than fifty novel NBS-LRRs have been characterized from several plant species. The miR482/2118 superfamily has been demonstrated to suppress a wide range of *R* genes, conferring resistance to fungal, bacterial and viral pathogens. Understanding the detailed mechanism by which miRNAs target NBS-LRRs is needed in order to engineer pathogen resistance using NBS-LRR genes in tomato plants.

## Author Contributions

S-QO and PL designed the experiments. S-QO wrote the paper. PL contributed to data analysis and interpretation. KB contributed to design this project and revised this manuscript. H-MJ and MZ performed the experiments in concert with YG, X-XC, H-YM, YZ, and W-YF who prepared the materials and total RNA extraction. All authors read and approved the final manuscript.

## Conflict of Interest Statement

The authors declare that the research was conducted in the absence of any commercial or financial relationships that could be construed as a potential conflict of interest.

## Footnotes

1 https://solgenomics.net/

## Supplementary Material

FIGURE S1 Original Northern blot result of slmiR482a.

FIGURE S2 Original Northern blot result of slmiR482b.

FIGURE S3 Original Northern blot result of slmiR482c.

FIGURE S4 Original Northern blot result of slmiR482d-3p.

FIGURE S5 Original Northern blot result of slmiR482d-5p.

FIGURE S6 Original Northern blot result of slmiR482e-3p.

FIGURE S7 Original Northern blot result of slmiR482e-5p.

FIGURE S8 Original Northern blot result of Solyc12g099060.

TABLE S1 Predicted targets of sly-miR482d family.
