# Supplementary figures and images for: FRG3, a Target of slmiR482e-3p, Provides Resistance against the Fungal Pathogen Fusarium oxysporum in Tomato
Source: Front Plant Sci. 2018 Jan 25;9:26. doi: 10.3389/fpls.2018.00026 (PMC5797444; doi:10.3389/fpls.2018.00026)

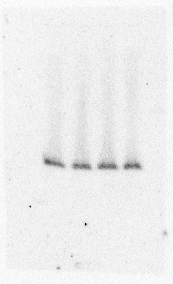

Supplement: FIGURE S1 — Original Northern blot result of slmiR482a. [file Image_1.TIF]

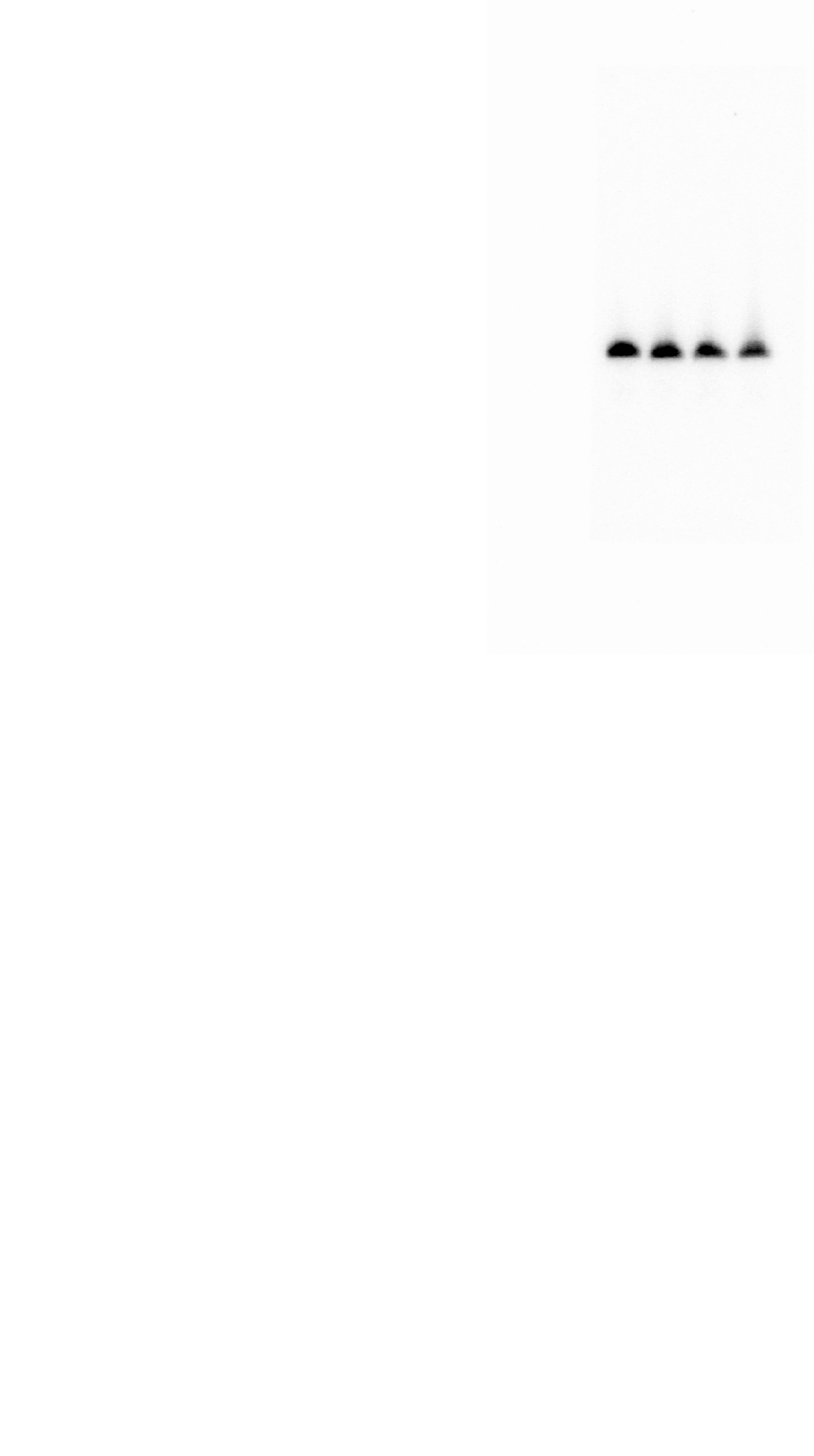

Supplement: FIGURE S2 — Original Northern blot result of slmiR482b. [file Image_2.TIF]

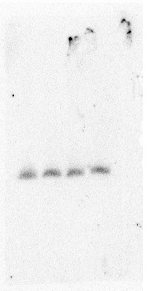

Supplement: FIGURE S3 — Original Northern blot result of slmiR482c. [file Image_3.TIF]

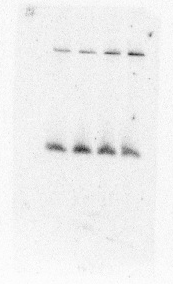

Supplement: FIGURE S4 — Original Northern blot result of slmiR482d-3p. [file Image_4.TIF]

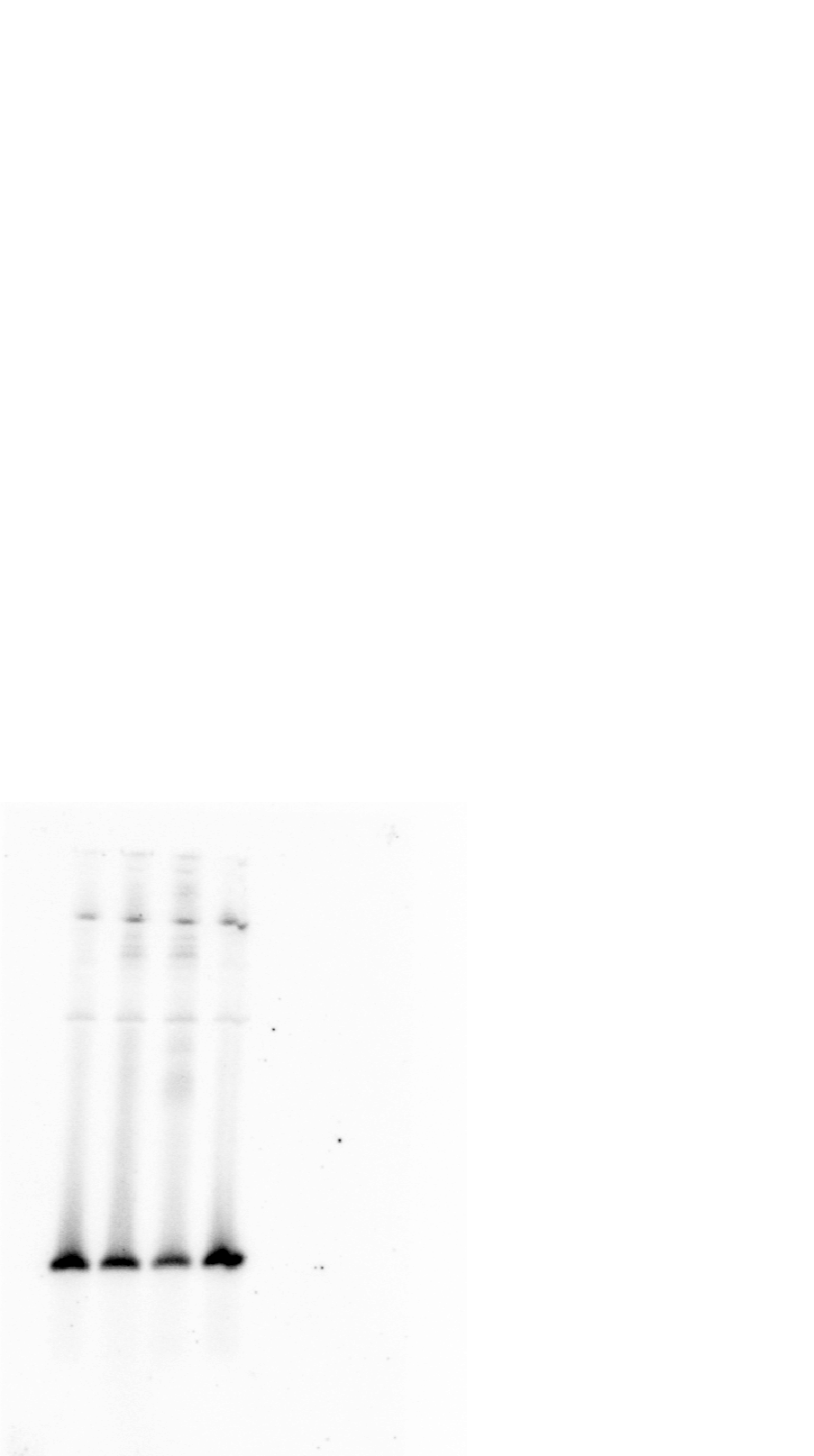

Supplement: FIGURE S5 — Original Northern blot result of slmiR482d-5p. [file Image_5.TIF]

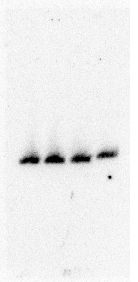

Supplement: FIGURE S6 — Original Northern blot result of slmiR482e-3p. [file Image_6.TIF]

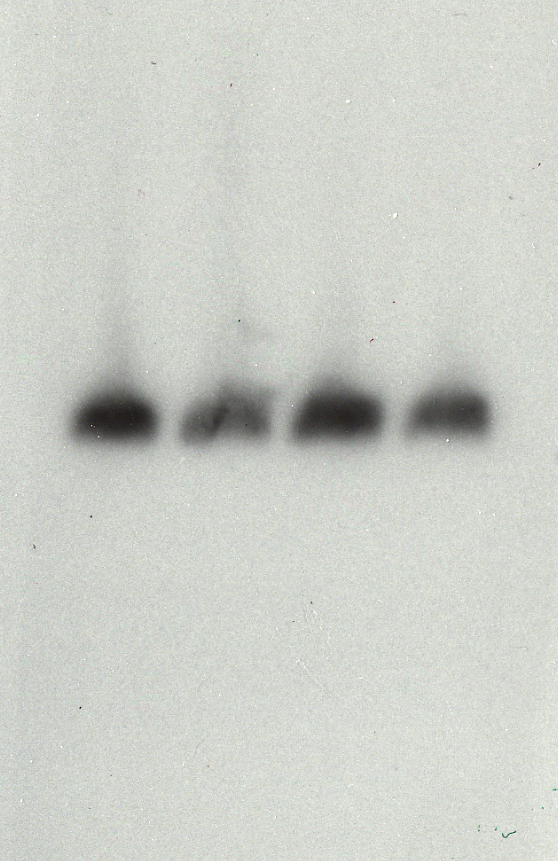

Supplement: FIGURE S7 — Original Northern blot result of slmiR482e-5p. [file Image_7.TIF]

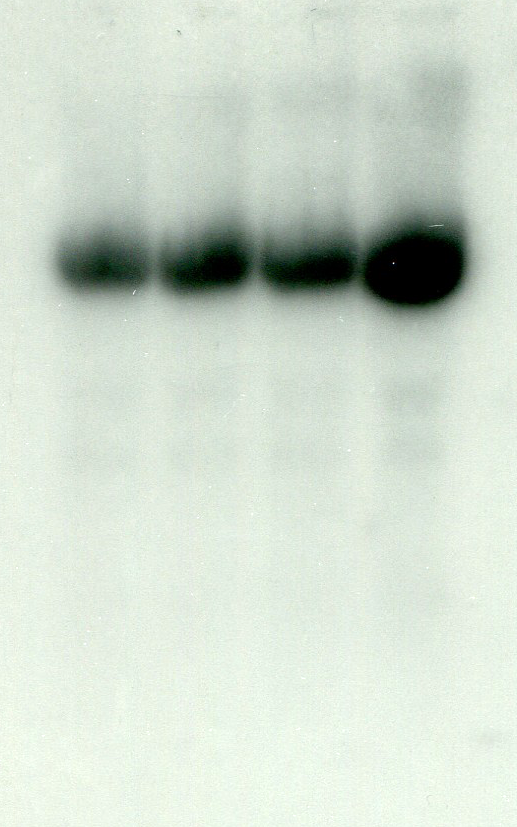

Supplement: FIGURE S8 — Original Northern blot result of Solyc12g099060. [file Image_8.TIF]
